# Properties of Mucoid Serotype 3 *Streptococcus pneumoniae* From Children in China

**DOI:** 10.3389/fcimb.2021.648040

**Published:** 2021-03-24

**Authors:** Ying Yang, Chun-Zhen Hua, Chao Fang, Yong-Ping Xie, Wei Li, Yong Fu, Feng Gao, Kai-Hu Yao

**Affiliations:** ^1^ Department of Infectious Diseases, The Children’s Hospital, Zhejiang University School of Medicine, National Clinical Research Center For Child Health, Hangzhou, China; ^2^ Department of Neurology, The Children’s Hospital, Zhejiang University School of Medicine, National Clinical Research Center For Child Health, Hangzhou, China; ^3^ Clinical Laboratory Center, The Children’s Hospital, Zhejiang University School of Medicine, National Clinical Research Center For Child Health, Hangzhou, China; ^4^ Department of Otolaryngology, The Children’s Hospital, Zhejiang University School of Medicine, National Clinical Research Center For Child Health, Hangzhou, China; ^5^ Department of Microbiology, Beijing Children’s Hospital, Capital Medical University, Beijing, China

**Keywords:** *Streptococcus pneumoniae*, mucoid colony, serotype 3, antimicrobial susceptibility, molecular epidemiology, sequence types, clonal complex

## Abstract

**Objective:**

To investigate the characteristics of hosts, antimicrobial susceptibility, and molecular epidemiology of mucoid serotype 3 *Streptococcus pneumoniae* (*S. pneumoniae*) isolated from children in China.

**Method:**

*S. pneumoniae* isolates collected between January 2016 and December 2019 were analyzed. *S. pneumoniae* isolates with mucoid phenotype were selected visually, and serotype 3 isolates were confirmed by Quellung reaction. The antimicrobial susceptibility was measured by E-test. Multilocus sequence typing was used for clonal analysis.

**Results:**

Twenty (3.04%) isolates of mucoid serotype 3 *S. pneumoniae* were identified from 657 clinical isolates, and all of them were noninvasive strains. The mean age of the hosts was 5.69 ± 3.28 years. The isolates included: 50.0% from the dissected tonsil or adenoid tissue in children with obstructive sleep apnea-hypopnea syndrome, 45.0% from sputum or bronchial lavages in children with pneumonia, and 5.0% from vaginal secretions of one patient with vulvovaginitis. All isolates were susceptible to penicillin, cefuroxime, ceftriaxone, meropenem, vancomycin, levofloxacin, trimethoprim/sulfamethoxazole, and rifampin but resistant to erythromycin. Sequence type (ST)505 and its clonal complex (CC) were the main genotypes (95%). Antimicrobial susceptibility of ST180 and ST505 were compared, and the minimum inhibitory concentration (MIC) of ST505 isolates was significantly higher than that of ST180 for tetracycline, trimethoprim/sulfamethoxazole, and meropenem.

**Conclusions:**

Mucoid serotype 3 *Streptococcus pneumoniae* can be isolated from various body parts, among which the respiratory system is the most common. It can cause noninvasive infection in children, and it has high susceptibility to a variety of antibiotics, especially β-lactams, but is resistant to macrolides. CC505 is the novel clonal complex found in China, which may be related to the worldwide mainstream clonal complex (CC180) but has its own biological characteristics.

## Introduction

*Streptococcus pneumoniae* (*S*. *pneumoniae*) is an important pathogenic microorganism worldwide, especially among children under 5 years old (Adam, 2002). It not only colonizes the human nasopharynx (Subramanian et al., 2019) but also causes invasive (Loughran et al., 2019) and noninvasive infections (Katz and Williams, 2018). Thus, *S. pneumoniae* poses a great threat to human health and has brought a heavy burden to patients and their families, and society (Wahl et al., 2018). Though there are over 95 serotypes of *S. pneumoniae*, different serotypes have different infection rates in humans (Hausdorff and Hanage, 2016). With the universal vaccination of Pneumococcal Conjugate Vaccine (PCV), the circulating pneumococcal serotypes have changed dramatically. There was a significant decrease in the serotypes covered by conjugate vaccines except for serotype 3 in both vaccinated and non-vaccinated individuals (Hausdorff and Hanage, 2016). In addition, the protection by PCV13 against serotype 3 *S. pneumoniae* does not seem to work as well as other serotypes, and many cases of serotype 3 infections in PCV13 immunized individuals have been reported (Oligbu et al., 2016; Silva-Costa et al., 2018; Goettler et al., 2020). There are many possible explanations for this unexpected finding, one of which is that the extremely thick capsule of serotype 3 may play an important role in avoiding antibody-mediated killing. Serotype 3 *S*. *pneumoniae* with thick mucoid capsule forms mucoid-type colonies *in vitro* (Dunne et al., 2010). Therefore, it is important to study the biological characteristics of mucoid serotype 3 *S. pneumoniae* in clinical settings. Serotype 3 is the second most common cause of invasive diseases in adults, which can lead to pneumonia, sepsis, and empyema/pleuritis. Previous studies have shown that mucoid serotype 3 *S. pneumoniae* is more virulent than other types, which causes higher mortality and longer hospital stay (Sugimoto et al., 2017). Contrary to adult patients, children rarely have infection with mucoid serotype 3 strain. Therefore, there is still much to know about its characteristics in pediatric patients (Nakano et al., 2016). Here, we have isolated 20 mucoid serotype 3 *S*. *pneumoniae* isolates from children in China and analyzed the characteristics of these patients, antimicrobial susceptibility, and molecular epidemiology of the isolates.

## Materials and Methods

### Patients and Clinical Isolates

This lab-based study of pediatric patients (under 18 years old) was conducted at Children’s Hospital, Zhejiang University School of Medicine between January 2016 and December 2019. All isolates of *S*. *pneumoniae* from patients with pneumococcal disease during this period were stored in a -80° C freezer. The isolates with mucoid colony morphology that were proved to be serotype 3 *Streptococcus pneumoniae* were enrolled in this study. If the isolates were separated from aseptic specimens (blood, cerebrospinal fluid, pleural fluid, ascites, joint puncture fluid, etc.), they were considered as invasive strains. If the isolates were separated from non-aseptic specimens (sputum, alveolar lavage fluid, vaginal secretions, surgically resected tonsils or adenoid tissues, etc.) and grew as a pure culture or as the dominant organism (Li et al., 2017), they were considered as noninvasive strains. Patients were divided into five different age groups for analysis: infant (0~1 years), toddler (1~3 years), preschooler (3~6 years), schoolchild (6~12 years), and adolescent (12~18years).

This study was approved by the ethics committees of Children’s Hospital, Zhejiang University School of Medicine (2019-IRB-136). Informed consent of children and their legal guardians were waived because this was a retrospective study.

### Reidentifying and Serotyping

The stored isolates were plated on Columbia blood agar plates containing 5% sheep blood. Bacteria were cultured in an incubator containing 5% CO2 at 35°C for 16-20 hours. Then the morphology of the colonies was examined. Reidentification of *S*. *pneumoniae* was performed by Optochin susceptibility test (diameter of bacteriostatic circle>14 mm). Mucoid isolates of *S. pneumoniae* were visually identified from all isolates on the basis of colony morphology described in the Manual of Clinical Microbiology, 10th edition (Spellberg and Brandt, 2011), then the serotypes of these mucoid isolates were identified using Quellung reaction with all available type-specific pneumococcal rabbit-antisera (SSI, Denmark). Positive Quellung reaction was defined as bacteria appear ‘swollen’ and more visible when the type-specific antibody binds to the capsule of the pneumococcus and leads to a change in its refractive index (Habib et al., 2014). Briefly, antiserum was added to a bacterial cell suspension from fresh overnight pure culture of pneumococci, and the capsule swelling reaction was observed with a differential phase microscope. Tests were carried out with individual antisera until a positive reaction was observed.

### Antimicrobial Susceptibility Tests

Antimicrobial susceptibility tests were performed using the E-test and the minimum inhibitory concentrations (MICs) method following instructions from the manufacture. Ten antibiotics were tested(μg/ml): penicillin (0.002~32), cefuroxime (0.016~256), ceftriaxone (0.002~32), erythromycin (0.016~256), tetracycline (0.002~256), meropenem (0.002~32), trimethoprim/sulfamethoxazole (0.002~32), rifampicin (0.002~32), vancomycin (0.016~256) and levofloxacin (0.002~32). The breakpoints used for interpretation were designed according to CLSI M100-S28 standard (Weinstein et al., 2018). ATCC 49619 was used as the standard control strain.

### DNA Extraction

DNA extraction was performed by boiling 200 μl of bacteria suspension for 10 min. Then the suspension was centrifuged at 12,000 rpm/min for 10 min. The supernatant containing genomic DNA was transferred to a new tube and stored at −20°C (Li et al., 2019).

### Multilocus Sequence Typing

Multilocus Sequence Typing (MLST) was performed using the primers of 7 housekeeping genes (*aroE*, *gdh*, *gki*, *recP*, *spi*, *xpt*, *ddl*) provided on the PUBMLST website (https://pubmlst.org), and the optimized polymerase chain reaction (PCR) was carried out with Platinum Taq polymerase from Hangzhou Nuoyang Biotechnology Co. Ltd (94°C for 5 minutes and 35 cycles of 94°C for 30 seconds, 50°C for 30 seconds and 72°C for 3 minutes). The positive products, which were confirmed by nucleic acid electrophoresis, were purified and sequenced by Beijing Qingke Biotechnology Co., Ltd. We had used the bidirectional sequencing method to identify the sequence type of each isolate and only confirmed the sequence type when the bidirectional sequencing results were consistent. The sequences were analyzed and compared to the standard sequences on PubMLST database to identify the sequence type (ST) for each isolate. STs that could not be found in the database were submitted to update the current database (Sousa et al., 2005). Analysis of the STs and assignment to clonal complex (CC) was performed using PHYLOViZ 2.0 program. (http://phyloviz.readthedocs.io/en/latest/#). The STs that shared at least five of seven allelic variants composed a CC. Single locus variants (SLVs), double locus variants (DLVs), and triple locus variants (TLVs) of a specific clone were identified. The minimum evolutionary tree was generated by MEGA 7 with a Neighbor-joining algorithm (Saitou and Nei, 1987; Kumar et al., 2016).

### Statistical Analysis

Continuous data with normal distribution were expressed as mean ± standard deviation ($\bar{x}$± *S*), while those with non-normal distribution were expressed by percentile (MIC_50_, MIC_90_, MIC_min_, MIC_max_). Categorical data were expressed as number (%). Categorical variables were compared using the χ2 test, and continuous variables were compared using the two-sided Mann-Whitney U nonparametric test between two groups. All statistical analyses were performed using Microsoft Excel 2007 and SPSS Statistics 20.0 software. A *P*< 0.05 was considered statistically significant.

## Results

### Characteristics of Hosts and Specimen Types

There were 657 isolates of *S. pneumoniae* during the study period, including 21 isolates with a mucoid colony. Further serotyping results showed that 20 isolates were serotype 3 and 1 isolate was serotype 37. Mucoid serotype 3 isolates accounted for 3.04% (20/657) of all pneumococcal isolates in this period. All 20 type 3 isolates were noninvasive and were isolated in 2016 (n=1), 2017 (n=5), 2018 (n=8), and 2019 (n=6), respectively. The ages of hosts ranged from 11 days old to 12 years and 11 months old, and the mean age was(5.69 ± 3.28)years old. The male to female ratio was 1.86(13:7). None of them had been vaccinated with PCV10 or PCV13. The sites of isolation included dissected tonsil or adenoid tissue in children with obstructive sleep apnea-hypopnea syndrome (OSAHS) (10 isolates, 50.0%), deep sputum (6 isolates, 30.0%), or bronchial lavages (3 isolates,15.0%) in pneumonia patients, and vaginal secretions in a pediatric patient with vulvovaginitis (1 isolate, 5.0%) (**Table 1**). After being treated with β-lactams antibiotics according to susceptibility test *in vitro*, all patients had improved their clinical conditions. The repeated bacterial culture of deep sputum of 9 patients with pneumonia, vaginal secretion of a patient with vulvovaginitis, and nasopharyngeal swab of 10 patients with OSAHS turned negative.

**Table 1:** Clinical characteristics of children with mucoid serotype 3 *Streptococcus pneumonia*.

	Infant (n = 3)		Toddler (n = 1)	Preschooler (n = 5)	Schoolchild (n = 10)	Adolescence (n = 1)	Total (n = 20)
	n (proportion,%)		n (proportion,%)	n (proportion,%)	n (proportion,%)	n (proportion,%)	n (proportion, %)
**Gender**							
male		1(5.0)	1(5.0)	5(25.0)	5(25.0)	1(5.0)	13(65.0)
female		2(10.0)	0(0.0)	0(0.0)	5(25.0)	0(0.0)	7(35.0)
**Diagnosis**							
OSAHS^a^		0(0.0)	0(0.0)	3(15.0)	7(35.0)	0(0.0)	10(50.0)
pneumonia		3(15.0)	1(5.0)	2(10.0)	2(10.0)	1(5.0)	9(45.0)
vulvovaginitis		0(0.0)	0(0.0)	0(0.0)	1(5.0)	0(0.0)	1(5.0)
**Treatment^b^**							
Cephalosporins		3(15.0)	0(0.0)	4(20.0)	10(50.0)	1(5.0)	18(90.0)
Carbapenems		1(5.0)	1(5.0)	1(5.0)	0(0.0)	0(0.0)	3(15.0)
Glycopeptides		0(0.0)	0(0.0)	1(5.0)	0(0.0)	0(0.0)	1(5.0)
Macrolides		0(0.0)	0(0.0)	0(0.0)	3(15.0)	0(0.0)	3(15.0)
lincosamides		0(0.0)	1(5.0)	0(0.0)	0(0.0)	0(0.0)	1(5.0)
**Specimen Types**							
tonsil or adenoid tissue		0(0.0)	0(0.0)	3(15.0)	7(35.0)	0(0.0)	10(50.0)
deep sputum		3(15.0)	1(5.0)	2(10.0)	0(0.0)	0(0.0)	6(30.0)
bronchial lavages		0(0.0)	0(0.0)	0(0.0)	2(10.0)	1(5.0)	3(15.0)
vaginal secretions		0(0.0)	0(0.0)	0(0.0)	1(5.0)	0(0.0)	1(5.0)

^a^OSAHS, obstructive sleep apnea-hypopnea syndrome.

^b^Treatment, 6 patients were treated with two antibiotics.

### Antimicrobial Susceptibility

The susceptibility of 20 isolates to penicillin, cefuroxime, ceftriaxone, meropenem, vancomycin, levofloxacin, sulfamethoxazole, and rifampicin was 100%. The resistance rates to tetracycline and erythromycin were 70.0% and 100%, respectively (**Table 2**).

**Table 2:** Antimicrobial susceptibility of 20 *Streptococcus pneumoniae* isolates.

Antibiotics	MIC_min_ (μg/ml)	MIC_50_ (μg/ml)	MIC_90_ (μg/ml)	MIC_max_ (μg/ml)	S^a^ (%)	I^b^ (%)	R^c^ (%)
Penicillin	0.003	0.008	0.012	0.012	100.0	0.0	0.0
Cefuroxime	0.016	0.016	0.061	0.064	100.0	0.0	0.0
Ceftriaxone	0.003	0.004	0.012	0.016	100.0	0.0	0.0
Meropenem	<0.002	0.003	0.004	0.004	100.0	0.0	0.0
Vancomycin	0.25	0.38	0.5	0.75	100.0	0.0	0.0
Levofloxacin	0.38	0.5	0.73	0.75	100.0	0.0	0.0
Trimethoprim/sulfamethoxazole	0.016	0.047	0.064	0.064	100.0	0.0	0.0
Rifampicin	0.004	0.008	0.016	0.016	100.0	0.0	0.0
Tetracycline	0.064	12	16	16	30.0	0.0	70.0
Erythromycin	>256	>256	>256	>256	0.0	0.0	100.0

^a^S, susceptible.

^b^I, intermediate.

^c^R, resistant.

### Multilocus Sequence Typing

**Figure 1:**
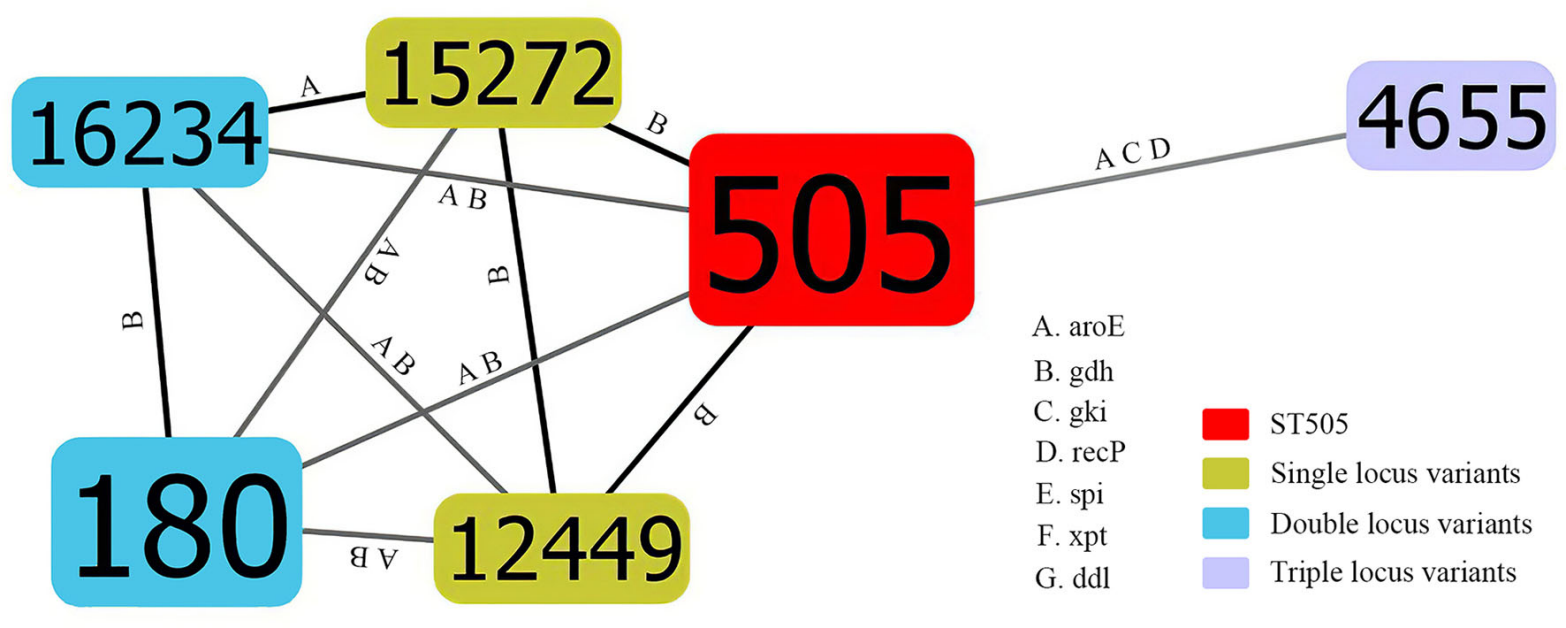
Population snapshot of mucoid serotype 3 *S. pneumoniae*. The STs were displayed as a goeBURST nLVs graph based on the allelic profiles. Each number represents one ST, and the area of each circle indicates the prevalence of ST in the MLST data of this study. All 6 STs were shown here and the different alleles between STs were presented. The letter A, B, C, D, E, F and G indicates *aroE*, *gdh*, *gki*, *recP*, *spi*, *xpt* and *ddl*, respectively. SLVs, DLVs and TLVs of ST505 were shown by different colors. MLST, Multilocus sequence typing; ST, Sequence type; SLVs, Single locus variants; DLVs, Double locus variants; TLVs, Triple locus variants.

**Figure 2:**
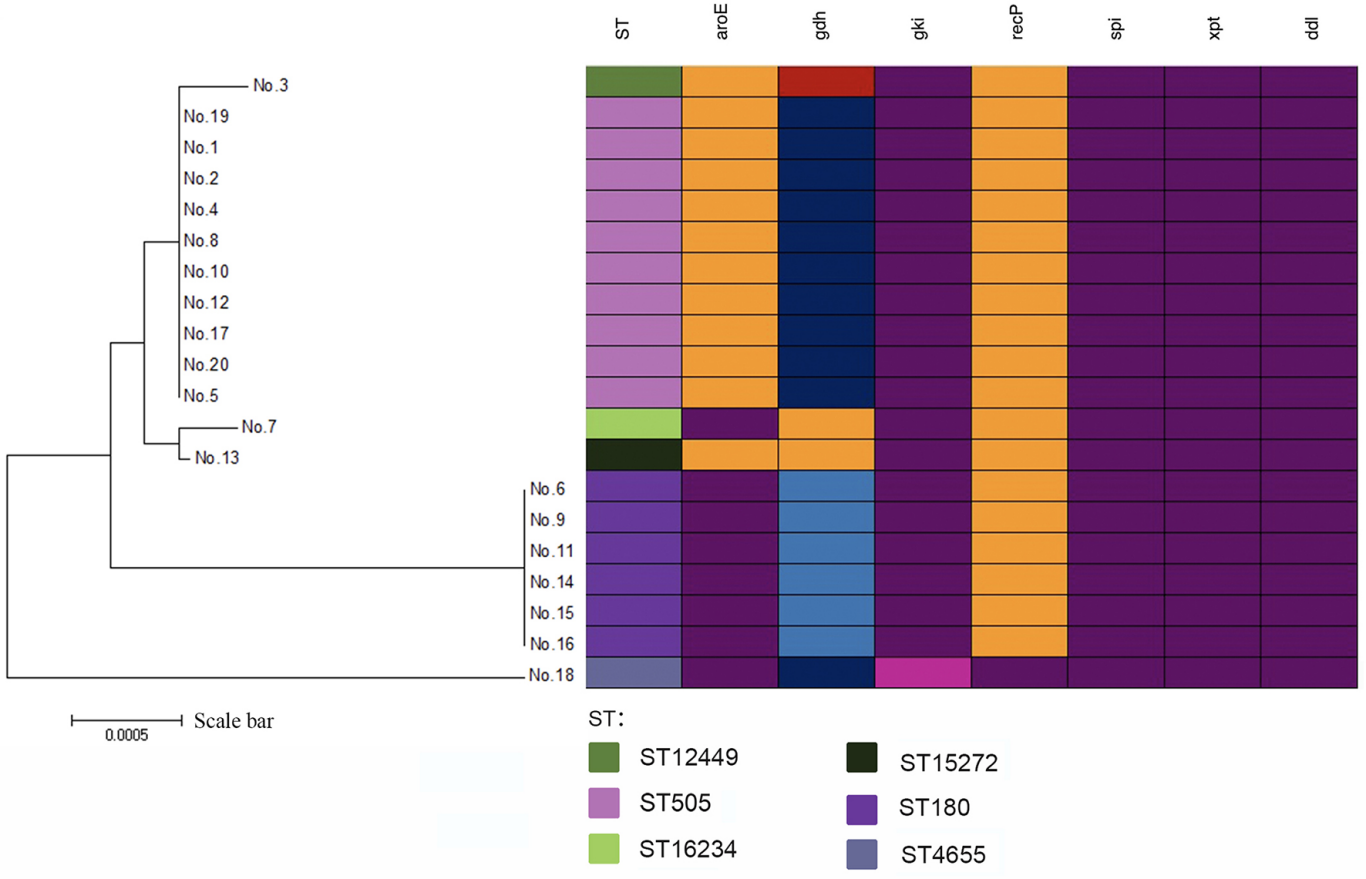
A minimum evolutionary tree of 20 mucoid serotype 3 *S. pneumoniae* isolates. The farther the straight distance was, the greater the gene sequence gap between isolates was. Mutations in alleles were shown by different colors.

There was a total of 6 STs identified by MLST analysis among 20 isolates. The most prevalent ST was ST505(50%,10/20). Other STs included ST180(30%,6/20), ST12449 (5.0%,1/20), ST15272(5.0%,1/20), and ST4655(5.0%,1/20). In addition, there was one newly identified ST (7-667-2-10-6-1-22), which had received an ST number (ST16234). There was one clone complex called CC505 with 95% (19/20) of the isolates being assigned to it. We found that ST505 was identified with two SLVs (ST12449, ST15272), two DLVs (ST180, ST16234), and one TLVs (ST4655) (**Figure 1**). According to the sequence variation of these seven housekeeping genes, the phylogenetic distance between these isolates was shown in the minimum phylogenetic tree. In addition, there was only one genotype of *spi*, *xpt*, and *ddl* in 20 isolates, while there were 4 genotypes *of gdh* and two genotypes *of gki*, *aroE* and *recP* (**Figure 2**).

Comparing the characteristics of ST505 and ST180, we found no significant difference in hosts’ gender, age, specimen types, and disease distribution (*P*>0.05). In terms of antimicrobial susceptibility, all ST505 isolates were resistant to tetracycline, while ST180 isolates were susceptible (*P*<0.05). All ST505 and ST180 isolates were both susceptible to trimethoprim/sulfamethoxazole, but the MIC of ST505 was significantly higher than that of ST180 (*P*<0.05). All ST505 and ST180 isolates were susceptible to β-lactams. There was no significant difference in the MIC of penicillin, cefuroxime, and ceftriaxone between the two groups (*P*>0.05). On the other hand, the MIC of meropenem in ST505 isolates was higher than that in ST180 isolates (*P*<0.05) (**Table 3**).

**Table 3:** Differences of clinical and microbiological characteristics between ST^a^505 and ST180.

	ST505 (n = 10)	ST180 (n = 6)	*P*
**Gender**	n (proportion,%)	n (proportion,%)	
male	6(60.0)	1(16.7)	0.145
female	4(40.0)	5(83.3)	
**Age**	n (proportion,%)	n (proportion,%)	
infant	2(20.0)	1(16.7)	0.178
toddler	1(10.0)	0(0.0)	
preschooler	1(10.0)	4(66.7)	
schoolchild	5(50.0)	1(16.7)	
adolescence	1(10.0)	0(0.0)	
**Specimen Types**	n (proportion,%)	n (proportion,%)	
tonsil or adenoid tissue	4(40.0)	3(50.0)	0.764
deep sputum	3(30.0)	3(50.0)	
bronchial lavages	2(20.0)	0(0.0)	
vaginal secretions	1(10.0)	0(0.0)	
**Diagnosis**	n (proportion,%)	n (proportion,%)	
OSAHS^b^	4(40.0)	3(50.0)	1.00
pneumonia	5(50.0)	3(50.0)	
vulvovaginitis	1(10.0)	0(0.0)	
**Antibiotics**	MIC_50_(MIC_min_~MIC_max_) (μg/ml)	MIC_50_(MIC_min_~MIC_max_) (μg/ml)	
Penicillin	0.008(0.003~0.047)	0.006(0.003~0.012)	0.181
Cefuroxime	0.0195(0.016~0.064)	0.016(0.016~0.064)	0.428
Ceftriaxone	0.005(0.003~0.012)	0.0035(0.003~0.016)	0.368
Meropenem	0.004(0.002~0.004)	0.002(0.002~0.003)	0.016
Vancomycin	0.38(0.25~0.5)	0.44(0.25~0.5)	0.492
Levofloxacin	0.5(0.38~0.5)	0.5(0.38~0.75)	0.263
Trimethoprim/sulfamethoxazole	0.0555(0.032~0.064)	0.032(0.016~0.064)	0.042
Rifampicin	0.008(0.006~0.016)	0.006(0.004~0.012)	0.118
Tetracycline	16(12~16)	0.5(0.064~4)	0.001
Erythromycin	>256(>256~>256)	>256(>256~>256)	1.000

^a^ST, sequence type.

^b^OSAHS, obstructive sleep apnea-hypopnea syndrome.

## Discussion

Serotype 3 *S. pneumoniae* has raised global attention in the post-PCV era, which became a new emerging public health issue (Azarian et al., 2018). The incidence of serotype 3 infection has increased in many countries, and it has become the most common serotype in some specific populations (Hausdorff and Hanage, 2016; Goettler et al., 2020). Serotype 3 *S. pneumoniae* persisted from pre-PCV7 to post-PCV13, and the incidence rate of serotype 3 did not decrease significantly post-PCV13 even though it is covered in the vaccine (Hausdorff and Hanage, 2016). The reason for the failure of immunoprophylaxis against serotype 3 *S. pneumoniae* has not been determined yet. Some studies have suggested that the carriage rate of serotype 3 *S. pneumoniae* might have played an important role: there was no significant decrease, but even an upward trend in the number of carriers (Azarian et al., 2018; Lewnard et al., 2019; Madhi et al., 2020). Others suggested that the special characteristics of type 3 capsular polysaccharide may also be an important factor (Choi et al., 2016). Serotype 3 *S. pneumoniae* synthesizes capsular polysaccharide (CPS) in a unique way, and it can release CPS during its growth. The released CPS interferes with antibody-mediated killing, resulting in vaccination failure (Choi et al., 2016). Because of its thick capsule, serotype 3 *S. pneumoniae* has a mucoid colony morphology, which is different from that of a typical pneumococcus. Thus, we analyzed the biological characteristics of mucoid serotype 3 *S. pneumoniae* isolates, hoping to provide additional information to understand this microorganism. Here, we presented clinical characteristics and molecular epidemiological data of mucoid serotype 3 isolates from children in China and showed that they possess unique characteristics in terms of isolation sites, invasiveness, antimicrobial susceptibility, and genotype.

In our study, mucoid serotype 3 *S. pneumoniae* was found in children of all ages, but it was more common in the schoolchild group. The mean age of children was more than 5 years old, which was older than the most common age for pneumococcal infection (Ogihara et al., 2015). We suspected that bacteria with a thicker capsule may be more resistant to host phagocytosis, even for the older children. Mucoid serotype 3 *S. pneumoniae* was isolated from a wide range of body parts in our study. The respiratory tract was the most common site where *S. pneumoniae* can cause acute or persistent infection. In this study, we found that they can cause acute pneumonia. Up to 20% of cases developed severe symptoms such as atelectasis or pleural effusion (Goettler et al., 2020), which needed other combinational therapy like bronchoscopy (Sugimoto et al., 2017). More interestingly, we found that they can also cause persistent infection. Previously, it was reported that *S. pneumoniae* was common in the culture of tonsillar or adenoid specimens of children with OSAHS (Dirain et al., 2017). Our study provided further confirmation that mucoid serotype 3 *S. pneumoniae* might be an important organism for the pathogenesis of OSAHS. It can stimulate the body to produce excessive inflammation factors and cause hyperplasia in the tonsillar or adenoid glands. Pathological hyperplasia leads to persistent hypertrophy of glands and the occurrence of OSAHS in children. In addition, mucoid serotype 3 *S. pneumoniae* was also isolated from the genitourinary tract, supporting that respiratory tract bacteria can be transmitted to the reproductive tract by children’s hand-mouth movement to cause a secondary infection (Sikanic-Dugic et al., 2009). No patient in this study has ever been vaccinated with PCV because it is not a mandatory vaccine in China. Nonetheless, Hangzhou has entered the post-vaccine era in recent years with the popularization of vaccines and economic development. Thus, these isolates were from the post-PCV scenario, which represented the epidemiological characteristics of the non-vaccinated population in the post-PCV era but not the pre-PCV era. It has been reported that vaccination of PCV should have changed the epidemiological characteristics of both vaccinated and non-vaccinated individuals (Hausdorff and Hanage, 2016). Thus, we felt our study had provided some epidemiological reference value, though it can’t reflect the overall population characteristics in the post-PCV era.

With the increasing pressure of widely-used antibiotics in the world, the antimicrobial susceptibility of *S. pneumoniae* has declined sharply both in children and adults. It is interesting that mucoid serotype 3 *S. pneumoniae* seemed to go the opposite way. We suspected that the thick capsule in mucoid strain has an important protective effect on bacteria to be free from the selective pressure of external antibiotics, and a similar finding was discovered in mucoid *Pseudomonas aeruginosa* isolates (Ciofu et al., 2001). The latest surveillance showed that the resistance rate for *S. pneumoniae* to penicillin and erythromycin was 32.0% (12.1-51.9%) and 94.4% (90.7-98.1%), respectively, among children in mainland China (Fu et al., 2019). In this study, we tested the susceptibility of serotype 3 isolates for ten common antibiotics and found that mucoid serotype 3 *S. pneumoniae* had the following characteristics. First, it was highly susceptible to a variety of β-lactams. The MIC_50_ of penicillin was as low as 0.008 μg/ml, while the MIC_50_ of ceftriaxone was as low as 0.004 μg/ml. This was consistent with the susceptibility of most serotype 3 *S. pneumoniae* globally (Sugimoto et al., 2017), with the exception that some invasive isolates had shown a penicillin-resistant pattern now (Ubukata et al., 2020). Secondly, most isolates of *S. pneumoniae* in China were resistant to sulfonamides, with a rate of up to 74.4% (64.5–84.4%) (Fu et al., 2019). Nevertheless, mucoid serotype 3 *S. pneumoniae* was highly susceptible to it. Finally, all isolates showed high-level resistance to macrolides, and the MIC was more than 256 μg/ml. All the children had good clinical response after treatment with β-lactams antibiotics, and their bacterial culture turned negative, which provide additional proof that mucoid serotype 3 isolates were highly susceptible to β-lactams. However, the *in vivo*–*in vitro* paradox of macrolides has recently been reported. Many azithromycin-resistant pneumococcal pneumonia cases have been successfully treated using azithromycin alone or in combination (Kohno et al., 2014; Namkoong et al., 2016). Based on the antimicrobial susceptibility pattern of mucoid serotype 3 *S. pneumoniae*, β-lactams are the first choices for the treatment of infections. Macrolides can be considered as concomitant administration for serious cases.

We showed that ST505 and its clonal complex were the most common genotype of mucoid serotype 3 *S. pneumoniae* from children in this study. All the isolates were close to each other in both biological and genetic characteristics, and 95% of isolates belonged to CC505. In addition, we found that *spi*, *xpt*, and *ddl* were relatively conserved, while *gdh* was relatively variable. CC505 showed significant differences in its biological characteristics when being compared to the mainstream genotype in the world (CC180). First, CC180 is the most prevalent type of serotype 3 *S. pneumoniae* in the world, which has a long history in the world. Its main characteristic is that it has high virulence and often causes invasive infection (Inverarity et al., 2011). ST180 was reported as the only ST associated with the worst outcome in a large study performed in Scotland (Inverarity et al., 2011). However, all CC505 isolates in this study only caused noninvasive infection, suggesting that there might be a difference in virulence between CC505 in China and CC180 in the world, which needs to be confirmed in future studies. Secondly, the resistance of CC180 is typically limited to macrolides, which is also called international resistance clone Netherland^3^-31 (Echaniz-Aviles et al., 2019). However, CC505 in this study showed decreased susceptibility to other antibiotics besides macrolides. Thirdly, CC180 is popular all over the world and had been reported many times, including a recent publication in the Chinese population (Azarian et al., 2018; Li et al., 2019). CC505 was rarely reported in the world (Porat et al., 2008), and this was the first report of this clone in the Chinese population. In the era of post-PCV, there are more and more new clonal complexes of serotype 3 reported in the world, such as CC4909, CC260, CC5292, and CC458 (Echaniz-Aviles et al., 2019). Though CC505 and CC180 were so distinct, these two lineages might be genetically related because they share some common housekeeping genes. A similar phenomenon had been found between CC4909 and CC180. We suspected that CC505 might be originated from CC180 but have changed some biological characteristics during this process.

We analyzed the clinical and microbiological differences between ST505 and ST180 identified in our study, which were the top two large genotypes in this study. We found that they had differences in antibiotic resistance. First of all, an obvious difference in tetracycline resistance was identified. All ST505 isolates were resistant to tetracycline, while all ST180 isolates were susceptible to tetracycline. In addition, although the MIC of both ST505 and ST180 to sulfonamides and β-lactams were in the susceptible range, ST505 were less susceptible to sulfonamides and some β-lactams (meropenem) than ST180. Therefore, ST505 had higher antimicrobial resistance than ST180, which indicates that they may have distinct drug-resistance genes. There was even a multi-drug resistant ST505 clone reported (Porat et al., 2008).

There are some limitations in our study. First, we did not have the whole genome sequences of the isolates, which might provide more important information for the novel clonal complex (CC505) in China. In addition, information about antimicrobial-resistant genes, virulence factors, and capsule genes can be obtained from the whole genome sequences, which will provide better explanations of its microbiological characteristics. Therefore, future research has been planned to address these questions. Secondly, the data in this study were from a single Children’s Hospital, so the sample size was relatively small. To confirm the findings reported in this manuscript, we will conduct a multi-center study in the future. Thirdly, this study presented the data of the mucoid serotype 3 *S. pneumoniae* from non-vaccinated children in China in the post-PCV era. Thus, it only provided limited epidemiological reference value.

In conclusion, mucoid serotype 3 *Streptococcus pneumoniae* can be isolated from various parts of children’s bodies, among which the respiratory system is the most common. It can cause noninvasive infection in children, and it has high susceptibility to a variety of antibiotics, especially β-lactams, but is resistant to macrolides. CC505 is a novel clonal complex found in China, which may be related to the worldwide mainstream clonal complex (CC180) but has its own biological characteristics.

## Data Availability Statement

The raw data supporting the conclusions of this article will be made available by the authors, without undue reservation.

## Ethics Statement

The studies involving human participants were reviewed and approved by the ethics committees of Children’s Hospital, Zhejiang University School of Medicine. Written informed consent from the participants’ legal guardian/next of kin was not required to participate in this study in accordance with the national legislation and the institutional requirements.

## Author Contributions

YY collected the clinical data, performed the MLST analysis, and wrote the draft. C-ZH designed the study, performed the analysis, and critically reviewed the manuscript for important intellectual content. CF performed the antimicrobial susceptibility test with the E-test method. K-HY performed the serotyping test for all pneumococci isolates. Y-PX, WL, YF, and FG collected the clinical or laboratory data and revised the manuscript. All authors contributed to the article and approved the submitted version.

## Conflict of Interest

The authors declare that the research was conducted in the absence of any commercial or financial relationships that could be construed as a potential conflict of interest.
